# *In vitro* micro-physiological model of the inflamed human adipose tissue for immune-metabolic analysis in type II diabetes

**DOI:** 10.1038/s41598-019-41338-3

**Published:** 2019-03-20

**Authors:** Patthara Kongsuphol, Shilpi Gupta, Yunxiao Liu, Sajay Bhuvanendran Nair Gourikutty, Subhra K. Biswas, Qasem Ramadan

**Affiliations:** 1 Institute of Microelectronics, Agency for Science Technology and Research (A*STAR), 2 Fusionopolis Way, #08-02, Innovis, Singapore, 138634 Singapore; 2 Singapore Immunology Network, A*STAR, 8a Biomedical Grove, Immunos, Singapore, 138648 Singapore

## Abstract

Chronic inflammation mediated by the interaction of immune cells and adipocytes is a key underlying factor in obesity-associated type 2 diabetes mellitus (T2DM). Therefore, methods to investigate adipocyte-immune cells interaction and their immuno-metabolic status in obese/T2DM subjects not only serve as an early indicator of disease development but also provide an insight into disease mechanism. A microfluidic-based *in vitro* model of the human adipose that is interfaced with a co-culture of immune cell has been developed for *in vitro* immune-metabolic analysis. This miniaturized system integrates a biologically active *in vitro* cellular system within a perfusion-based microfluidic device for mimicking the major processes that characterize the interaction of adipose tissue with immune cells. A viable immune competent model of the adipocytes/PBMCs co-culture has been demonstrated and characterized. Our testing results showed that the inflammatory cytokine profile obtained from the on-chip culture agrees with those from static transwell based co-culture with more intense responses observed in the chip-based system. The microfluidic chip also allows time-resolved measurement of cytokines that provide reliable data and detailed mechanisms of inflammation. In addition, glucose uptake by the adipocytes from the chip-based cultures showed correlated insulin responsivity/resistivity to the expression of the cytokine profile in different dynamic culture conditions. Testing of the known diabetic drug, metformin, and neutraceutical compound, omega-3, on-chip show agreeable results as compared to the previously reported data. This organotypic culture system offers a physiologically relevant model that exhibits a key characteristic of type 2 diabetic adipose tissues and can be used to study the T2DM mechanisms and diabetic drug screening.

## Introduction

Adult-onset diabetes mellitus or type 2 diabetes mellitus (T2DM) is a global pandemic that affects more than 400 M people worldwide and is reported to be the direct cause of death to ~1.2 million in 2012 (10 facts on diabetes: http://www.who.int/features/factfiles/diabetes/en/). T2DM Patients exhibit insulin resistance condition that reduces glucose uptake into the tissues. The non-absorbed glucose leaves high glucose content to remain in the bloodstream. Prolong high blood glucose or hyperglycemia damages vascular system that subsequently leads to multiple complications, e.g. cardiovascular diseases^1^, blindness^2^, gangrene^3^, etc.

T2DM is tightly associated with obesity or excess body fat^4,5^. Recent studies have identified chronic inflammation as a common underlying factor for obesity, insulin resistance (IR) and T2DM^6^. Indeed, obese tissues are heavily infiltrated by inflammatory immune cells (e.g. monocytes, macrophages, Th1 cells) which interact with adipocytes to trigger chronic inflammation which blocks insulin action on adipocytes (i.e. IR) leading to T2DM^6–8^. Mice studies have shown immune cells to mediate chronic inflammation in adipose tissues thereby turning adipocytes into insulin resistant and T2DM. In addition, studies from the adipocyte-immune cells interaction have reported upregulation of pro-inflammatory cytokine gene expressions particularly in insulin resistive mice models^8–10^.

In human adipose tissue, different gene expression profiles were reported in obese and lean subjects^11–14^. Human adipose tissues express a number of inflammation-related genes^12,15^ with obese subjects displaying higher expression of these genes as compared to non-obese subjects^14^. Reduced infiltration of macrophage in adipose tissue was reported in surgery-induced weight loss subjects^12^. In addition, the study of peripheral blood showed higher expression of inflammatory biomarkers in T2DM as compared to prediabetes subjects^16^. These findings support the growing importance of immune-metabolic interplay in human obesity that link to insulin resistance and T2DM.

Owing to the importance of immune component in driving obesity-related T2DM, studying the dynamic interaction between the adipose tissue and the resident immune cells would provide an insight into the pathogenesis of the disease. The vast majority of basic research on T2DM, including adipocyte-immune cell interaction, has been conducted in animal models. Genetically modified or high-fat diet-induced animals that develop obesity and hyperglycemia are employed as surrogate models to understand T2DM in human^17–19^. While these models provide systemic *in vivo* settings, they are costly, difficult to handle, consume long breeding time, and more importantly, do not recapitulate the human physiology^20–22^. Despite the acquired knowledge from animal models, in particular, discoveries that sometimes mimic human T2DM mechanisms, many details of human T2DM pathogenesis including, adipocyte-immune cell interaction, still unclear, which limits therapeutic options. In addition, recent data obtained from human have raised concern regarding inter-species differences at various levels and the impact of these differences on the clinical translation of animal research findings^20^. This is a necessity considering the differences between animal and human immune responses and outcomes in preclinical models versus clinical trials.

Significant research efforts are currently focused on finding methods to transform drug screening from a system reliant on high-dose animal models to one based primarily on human-relevant *in vitro* models. To date, there are no physiologically relevant models illustrating the adipose-immune system interaction that could lead to accurate T2DM extrapolation. To obtain responses that closely represent human physiology, primary human cells (adipocytes or immune cells) culture in static systems (e.g. tissue culture plates) are used. However, these culture systems lack a dynamically controlled cell microenvironment^23^ therefore, provide only little biological relevance^24–27^. Conventional cell culture systems also require a large amount of cells, reagents and culture media. In addition, repetitive cultures must be carried out for snap-shot measurements of cell responses at different time points. These make static culture a rather expensive approach.

Furthermore, misinterpretation may arise from the snap-shot measurements as different groups of cells may react differently to the treatments and take different times to respond to the specific conditions^28^. A serious limitation for patient-derived samples is the minute quantity samples, which is often difficult to analyze using conventional cell culture or detection tools. Investigating the interaction between the crucial players of T2DM pathogenesis, the immune cells and adipocytes, and how this modulates their immune-metabolic status in obese or obesity-associated T2DM subjects not only provide insight into molecular mechanisms of this disease but also serve as an indicator of disease progression. Therefore, there is a real need for *in vitro* models which can closely mimic the physiological processes and predict the impact of the drug with an acceptable level of flexibility, accuracy, and reproducibility. Such models would significantly reduce experimentation expenses and speed up the screening processes.

**Figure 1:**
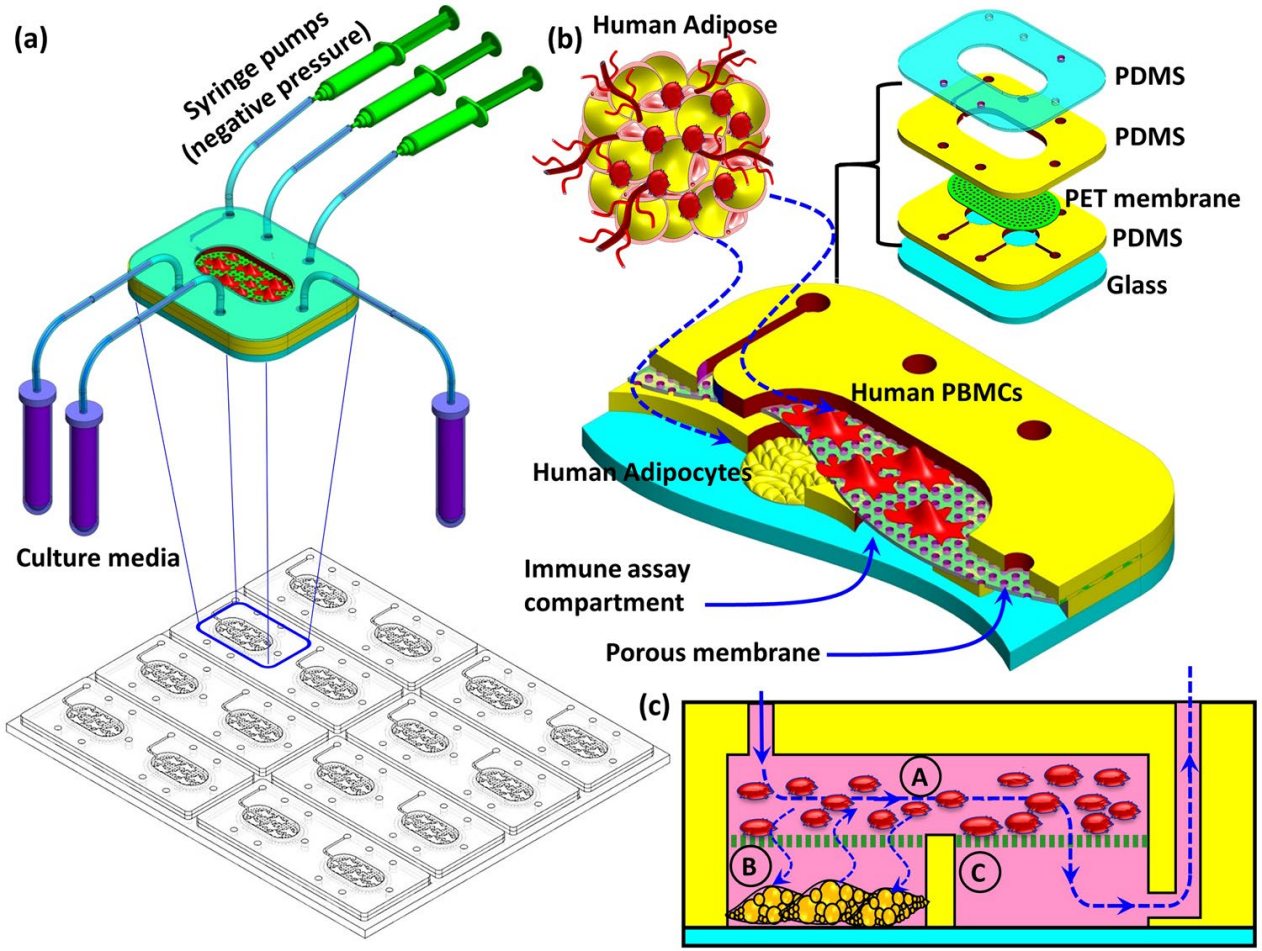
Schematic drawing of the adipocyte-immune cell interaction biological model within a microfluidic cell co-culture device. (**a**) Drawing of 8 microfluidic chips, each chip contains 2 cell culture units. Enlarged view of a single culture unit with fluidic connection ports. (**b**) Enlarged cross-sectional drawing of the microfluidic chip. To mimic the human adipose tissue, adipocyte and immune cells (peripheral blood mononuclear cell, PBMC) were co-cultured on a microfluidic chip. Adipocytes were cultured in the lower compartment whereas the PBMCs were cultured in the upper compartment of the chip. The multilayer structure of the chip is shown in the inset (**c**) Cross-sectional view of the microfluidic chip displaying the three compartments and microfluidic path: (A) PBMC culture compartment, (B) adipocyte culture compartment and (C) immunoassay compartment where samples are collected for immunoassay analysis. This allows sample collection without disrupting the cell culture thus enabling sample collection at different time points throughout the culture period. Arrows show the fluidic path and diffusion through the chip’s compartments.

Microfluidic-based cell culture techniques can potentially narrow the gap between *in vivo* and *in vitro* systems by providing a controlled cell microenvironment and integrating various cellular systems with enhanced cell-cell interactions^29–31^. To study the interaction between the adipocytes and immune cell related to T2DM, we have developed a microfluidic-based cell culture system for dynamic co-culture and communication (Fig. 1). This miniaturized system integrates a biologically active *in vitro* cellular system within a perfusion-based microfluidic device. This setting aims at mimicking the major processes that characterize the interaction of adipose tissue with immune cells in a single self-contained microfluidic system which enables time-resolved monitoring of cell responses to stimuli and simultaneous detection of a panel of inflammatory biomarkers as well as glucose uptake. The inter-organ cross-talk remains a critical area of human T2DM research that has been lacking thus far due to the lack of appropriate tools to perform such human tissue studies. The short-term implication of our proposed multi-compartment 3D microfluidic system is the ability to evaluate systemic interactions between human cells and understand the net effects of external stimuli in the context of T2DM. Our proposed model would complement existing rodent pathophysiological models of T2DM and increase the scientific rigour and applicability of findings initially discovered in rodent system to human T2DM and provides a step forward in bridging the existing translational gap between *in vivo* and *in vitro* studies.

## Results and Discussion

### Microfluidic chip and fluidic characterization

The 3D-microfluidic chip was fabricated to host the adipocyte-immune cell co-culture model. The chip comprises two (upper and lower) fluidic layers separated by a porous membrane (Fig. 1a–c). The lower part, which was bonded to a glass substrate, comprises two fluidic compartments (Fig. 1c). The upper compartment (A) spans across the two lower compartments (B and C) and used to host immune cell culture whereas the two lower compartments were used to host the adipocyte culture (B) and the downstream immunoassay (C). This assembly results in three fluidically connected compartments (Fig. 1c) through which the liquid continuously perfused at the desired flow rate.

Under physiological condition, the adipocyte experience only minimal interstitial shear stress of less than 0.01 Pa^32^. To maintain minimal shear stress on adipocyte, perfusion was introduced to the upper compartment (A) (Fig. 1c) and exit via lower compartment (C) to avoid direct flow to adipocyte. Using similar configuration of this perfusion setup, it was found in our previous study that application of flow speed at 15 nL/sec affected lipid droplet accumulation^28^, therefore lower perfusion flow rate of 10 nL/sec was used throughout the experiments. To evaluate the resultant shear stress onto the adipocyte membrane due to the media perfusion, the fluid velocity profile was analyzed using Finite Element Methods (COMSOL MultiPhysics, Sweden). It was found that fluid flows with highest velocity at the mid-height portion of compartment (B) (Supp. Fig. 1b) while the rest of the cell culture chamber exhibit relatively even distribution of low flow rate that produced minimal shear stress of only ~1 × 10^4^ dynes/cm^2^ (equivalent to 1 × 10^−5^ Pa) which was less than the interstitial stress. Thus perfusion speed of 10 nL/sec was chosen throughout the experiment.

To further ensure dynamic fluidic crosstalk between the three compartments, the lower compartment (B), which is designated for adipocyte culture, was filled with dyed PBS solution while the other compartments were filled with clear (undyed) PBS (Supp. Fig. 1c). After 18 hr of perfusion at 10 nL/sec, the coloured solution nearly vanished from the compartment B (Supp. Fig. 1a,c). This indicates that the dye diffused into the upper compartment (A) and washed out by perfusion through the outlet in the compartment (C). Meanwhile, the clear PBS diffused to the lower compartment (B) and slowly replaced all the dyed solution in the compartment. These observations confirmed the desired fluidic crosstalk between the three compartments through the porous membrane.

### Adipocyte & PBMC growth and viability on chip

**Figure 2:**
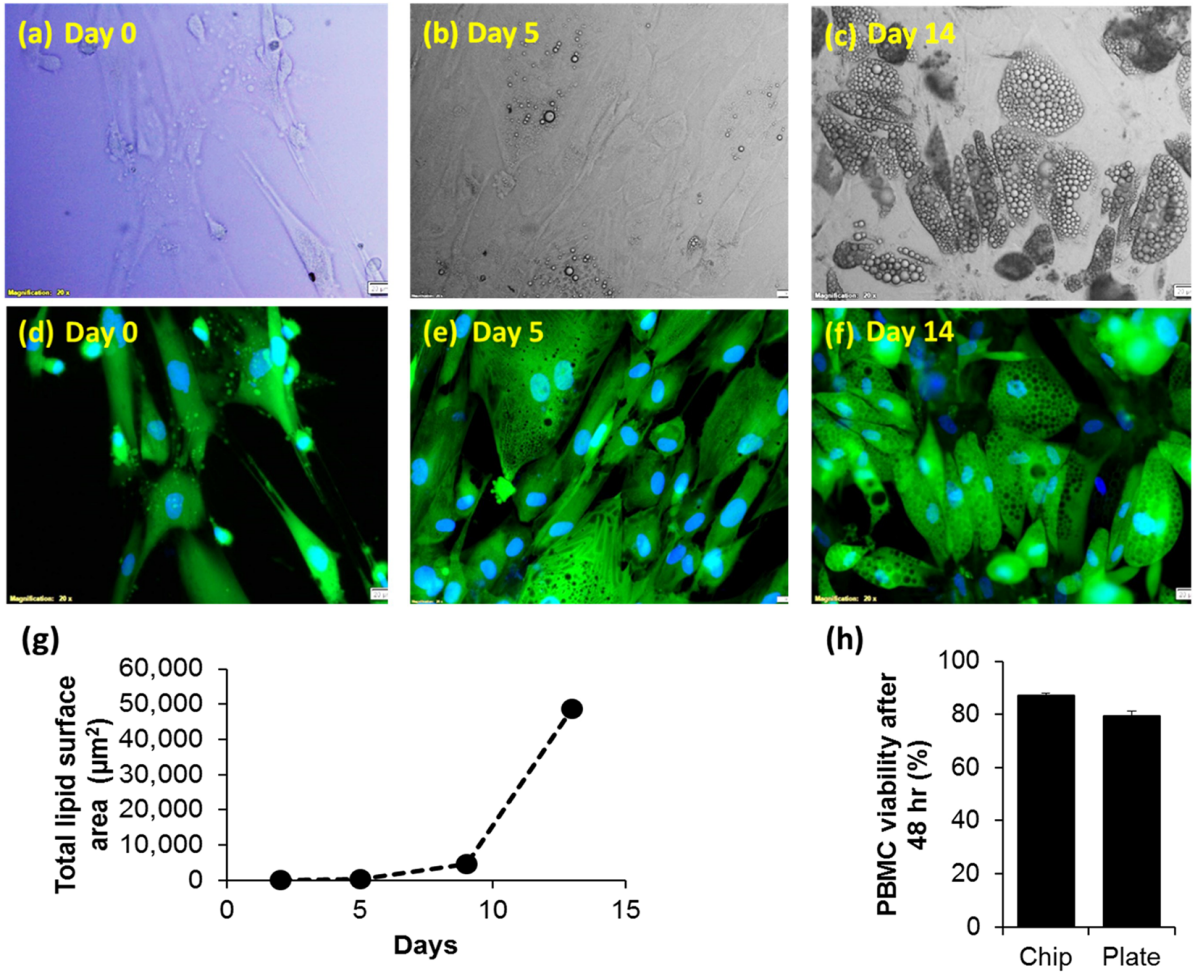
Adipocytes mono-cultured on-chip. (**a**–**c**) Bright field images (upper row) (**d**–**f**) Fluorescence images (lower row) of adipocyte differentiation sequence at 0, 5 and 14 days, respectively. The fluorescence images (**d**–**f**) show the stained intracellular content and nuclei and of adipocyte. All images were taken at 20x magnification; scale bars indicate 20 µm. (**g**) Total lipid covering area analyzed from bright field images at different days and (**h**) PBMCs viability cultured on-chip and transwell plate for 48 hr.

To ensure high viability of cells grown on-chip, monoculture of adipocytes and PBMCs were initially examined separately. Human Pre-adipocytes (HPAd) were cultured in the compartment (B) of the chip (Fig. 1c) until confluence. Perfusion with a flow rate of 10 nL/s was constantly applied to the upper compartment to supply fresh nutrients to the cells via the porous membrane. Fresh adipocyte differentiation medium was perfused into the chip to induce differentiation (adipogenesis). Cells growth and morphology were regularly monitored under the microscope. HPAd reached confluence ~3–4 days after seeding, and lipid droplets could be observed as early as 2 days after introducing the differentiation medium (data not shown). Droplet size (hypertrophy) and number (hyperplasia) gradually increased over the 14 days of differentiation period (Fig. 2a–c). While pre-adipocytes appeared flat without detectable lipid droplet (Fig. 2a), the differentiated adipocytes can be characterized by the presence of different sizes of lipid droplets (Fig. 2b,c). To better demonstrate cell boundary and lipid droplet accumulation, fluorescence staining of intracellular content was used as a marker to represent cell boundary. It is clearly shown in Fig. 2d–f that there was an increase in lipid droplets number (hyperplasia) at 5 and 14 days after differentiation. In addition, it was observed during capturing of florescence images that there were multifocal planes within the image at 14 days of differentiation, thus suggesting that cells grew in the vertical axis and formed a dome shape (Fig. 2f). In contrast to differentiated cells at 5 days (Fig. 2e) which demonstrated cell growth only in 2d configuration (increase in width and length), differentiated cells at 14 days show cell enlargement (hypertrophy) in 3d configuration (increase in width, length and height). Image analysis of total lipid area was also shown a proportional increase in the lipid area upon increase in differentiation days (Fig. 2g). These data confirm the differentiation of pre-adipocytes to adipocytes (adipogenesis) on-chip.

PBMCs were cultured in the upper compartment (A). Constant perfusion at a flow rate of 10 nL/s was applied directly to the the upper compartment throughout the culture period (Fig. 1c). After 48 hr of the culture, the cell viability was observed at ~87% (Fig. 2h) which is slightly higher than the viability of PBMC cultured on the static plate (~80%). This shows that PBMC can be maintained at high (>80%) viability when cultured dynamically on-chip.

### Dynamic cell culture on-chip VS static culture in transwell plate

#### Adipocyte-PBMC co-cultured on-chip and the effect of inflammation on the cell physical properties

**Figure 3:**
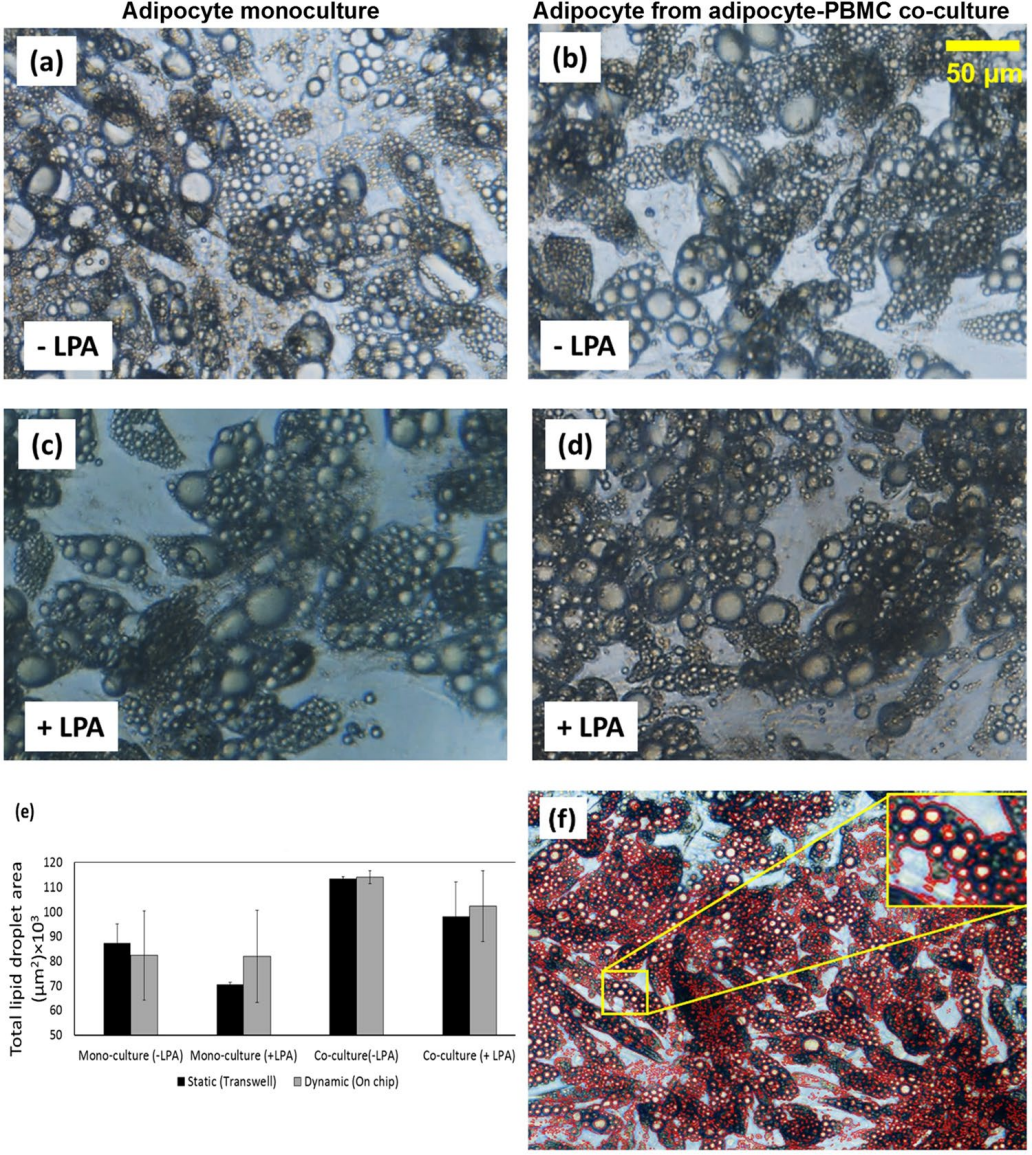
Bright field images of adipocytes which were cultured on-chip taken from different treatment conditions. (**a**) Untreated adipocyte monoculture, (**b**) untreated adipocyte from adipocyte-PBMC co-culture (**c**) 100 ng/mL LPA treated adipocyte monoculture and (**d**) 100 ng/mL LPA treated adipocyte from adipocyte-PBMC co-culture. (**e**) Total lipid droplet coverage area in both treated and untreated with LPA in static and dynamic culture. Total lipid surface area slightly increased in co-culture groups regardless of the LPA treatment. (**f**) Image of adipocyte highlighting the lipid droplets boundaries using CellSens software (Olympus, Japan).

Adipocytes were cultured in compartment B of the chip to fully differentiation, and PBMCs were then inoculated into the compartment A. To induce an inflammatory condition, cells were treated with100 ng/mL Lipid A from E. coli serotype R515 (LPA). Six different treatments conditions were conducted in parallel; these include: (i) adipocyte mono-culture with LPA treatment, (ii) adipocyte mono-culture without LPA treatment, (iii) PBMC mono-culture with LPA treatment, (iv) PBMC mono-culture without LPA treatment, (v) adipocyte-PBMC co-culture with LPA treatment and (vi) PBMC co-culture without LPA treatment. To examine the effect of the treatments/conditions, the cell size (hypertrophy) and lipid droplet population profile (hyperplasia) were regularly observed under a microscope and images were taken to estimate the total lipid surface area, as an indicator of the total lipid volume, using CellSens software (Olympus, Japan). It was observed that adipocytes maintained planar distribution even after proliferation and enlargement, therefore, the vast majority of lipid droplets were observed to form a monolayer (Figs 2a–f and 3a–d) with distingushable boundaries that allows the ustlization of image analysis to quantify the lipid surface coverage. Lipid droplets that were grown on top of the monolayer plane are of the minority population and were not considered for total lipid area analysis. An example of droplet analysis image is shown in Fig. 3f. The boundary of every lipid droplet within a single focal plane is automatically highlighted and the calculated area of the of boundary resembles the lipid surface. In general, cells in all treatment groups show a similar pattern of cell morphology, i.e. heterogeneous lipid droplet size (Fig. 3a–d). However, the data analysis from the 2D images taken from these different groups, show that the total lipid surface area slightly increased in co-culture groups regardless of the LPA treatment (Fig. 3e).

Similarly, no significant changes in droplet size were observed in the static cultures where cells also exhibited similar morphology in all treatment groups (Supp. Fig. S2). Adipose tissue enlargement is a consequence of the ability of adipocytes to store triglycerides as well as the ability of pre-adipocytes to differentiate into adipocytes (adipogenesis). It was reported that adipocyte treatment with Lysophosphatidic acid reduces triglyceride accumulation^33^. We observed significant fluctuation of lipid size droplet both in treated and untreated cell groups. Kim *et al*.^34^ reported that lipid-overloaded hypertrophic adipocytes are insulin resistant independent of adipocyte inflammation.

**Figure 4:**
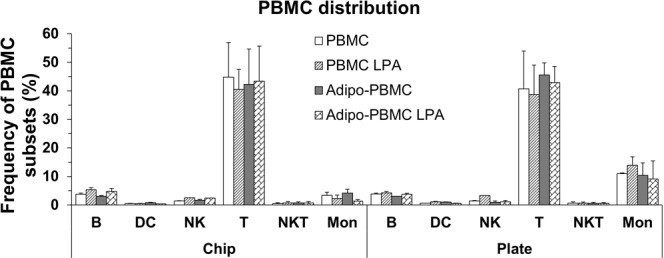
Distribution profile of PBMC cultured on chips and transwell plates from different treatment conditions. Data were represented as mean ± SEM of two independent experiments; however, frequency of NK cells of LPA treated-PBMC Set is from one experiment. Distribution profile of PBMC cultured on chips and transwell plates from different treatment conditions obtained by FACS.

Because PBMCs were cultured on the porous membrane, background pattern of the membrane pores interfered with the visibility of PBMC (Supp. Figs S3 and S4). Therefore, image analysis of PBMC could not be performed. PBMC were phenotyped using flow cytometric analysis. For the on-chip cell culture, data showed no significant difference in PBMC distribution in all the treatment groups (Fig. 4). The distribution of some key immune subsets in these PBMCs was as follows: B cells ~3–5%, NK cells 1.5–2.5%, T cells ~40–50%, NKT at ~0.5–0.7% and monocyte ~1–3%. When compared to the static cultures, the B, NK and T cell distributions show a similar pattern in both culture conditions (Fig. 4). However, monocytes displayed only ~1–3% distribution on-chip whereas they displayed ~9–13% distribution on transwell plate. The chip’s membrane was examined, and it was found that a substantial amount of PBMCs (~15% of total cells) were adhered to the membrane and could not be retrieved for FACS analysis. The adhered cells were stained and counted under the microscope. The results show that 70% of the cells displayed a positive signal for monocytes (Supp. Fig. S5). The total number of monocytes adhered to the chip membrane combined with those counted using FACS, give a total number of monocytes to be ~11–14% which is within the same range of the monocytes distribution observed in static culture suggesting a similar PBMC distribution pattern in both systems.

#### Immune status: detection of cytokines expression in the Adipocyte-PBMC co-culture

It has been well documented that immune cells infiltrate into the adipose tissue, interact with adipocytes and triggers signal activation and gene expression which is mediated by cytokine release^7^. It should also be noted that adipose tissue is recognized as an ‘immune organ’ which is considered as a source of inflammation^31,32^. Here, we monitored the release of three key pro-inflammatory cytokines IL-1β, IL-6 and TNF-α from the adipocyte and PBMC mono- and co-culture conditions upon presence or absence of the inflammatory agent, LPA. Adipocyte and PBMC mono- and co-cultures were carried out concurrently on the chip and transwell plate. Cells were treated for 24 hr and culture media were collected for cytokine measurement. Although the ratio of adipocyte: PBMC were similar in the chip and transwell plate cultures, the size of the culture compartments and media volume used were not proportioned. In addition, the perfusion applied to the on-chip culture further complicated the dilution of culture medium. Therefore, to compare cytokines secreted from the two cultured systems, the cytokines were normalized with the control cytokine baseline released from adipocyte monoculture without LPA treatment. Values are represented in fold change ± SEM.

**Figure 5:**
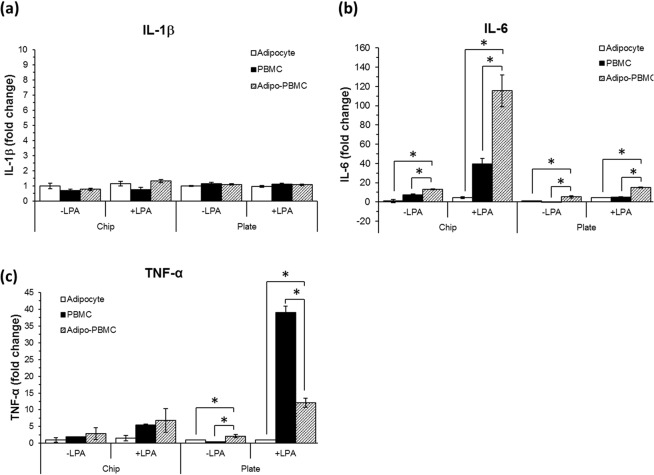
Comparison of pro-inflammatory cytokines (**a**) IL-1β, (**b**) IL-6 and (**c**) TNF-α released from dynamic (chip) and static transwell plate (plate) cultures at 24 hr of different treatment conditions. Data were represented as mean ± SEM of two independent experiments ran in duplicates. However, values of LPA-treated adipocytes are from one donor only. ONE-WAY ANOVA with Bonferroni correction post hoc test was used for statistical analysis. * Indicates statistical significance at *p* ≤ *0*.*05*. Each sample was run in triplicates.

IL- 1β shows no significant expression in all treatment groups (Fig. 5a). However, in both on-chip and transwell plate cultures increase in the levels of inflammatory cytokine, IL-6 was observed in adipocytes-PBMCs co-culture group when compared to adipocytes and PBMCs monoculture. These data suggest that immune cells and adipocytes cultured together fabricate inflammatory milieu. Upon comparing the on-chip and transwell plate cultures, IL-6 shows higher secretion in the on-chip cultures as compared to the transwell plate cultures (Fig. 5b). Furthermore, in both cultured systems, the co-culture cells show a higher amount of TNF-α secretion as compared to the adipocyte mono-cultures. Although no statistical difference was observed from mono- vs co-culture treatments in the on-chip cultures, clear tendency of increase in TNF-α is observed from the co-culture conditions as compared to the mono-cultured conditions (Fig. 5c).

We observed some notable differences upon comparing the response of cells under static and on-chip culture conditions, significantly higher level of IL-6 secretion was observed in dynamic on-chip LPA-treated PBMC-adipo co-culture set than those in the static co-cultures (Fig. 5b). This suggests that the increase in IL-6 secretion is not an additive effect of adipocyte and PBMC but a synergistic effect of the co-culture of both. Effect of LPA treatment on TNF-α levels was also observed to be different among the two culture conditions, wherein PBMC cultured under static conditions showed significantly higher levels of TNF-α as compared to on-chip PBMCs. This could be attributed to amplification of inflammatory circuit in a paracrine/autocrine manner via LPA-induced TNF-α. This effect can be particularly enhanced by the static culture which allows accumulation of TNF-α, therefore, stimulating more inflammation and enforcing more TNF-α secretion^35,36^.

Nevertheless, the LPA treated co-culture cells on the transwell plate show much less TNF-α secretion as compared to LPA-treated PBMC mono-culture. We speculated that this might be due to the interactive role of adipocyte and PBMC in which both of the cells exhibit immunoactive functions that may counterbalance each other activity thus resulting in a reduction of TNF-α released from PBMC. Irrespective of the extreme TNF-α value from PBMC mono-culture on transwell plate which may be caused by the static culture artefact (as mentioned above), it can be suggested from these experiments that both the on-chip and transwell plate cultures recapitulate relatively similar trends of cytokine response for the different treatments. It is evident from our experiments that IL-6 shows higher secretion in the on-chip cultures which is encouraging given its key role in insulin resistance and metabolic disease^35^. In addition, the dynamic on-chip cultures minimized the high TNF-α secretion as observed in the static culture, which may reflect a more physiologic dynamic state as encountered in the tissues. We speculate that the present on-chip setup possibly provides a more physiologic and dynamic interaction between stimuli and cells as well as between interacting cells (co-cultures) which are enhanced by perfusion.

Despite the fact that our compartmentalized fluidic system does not allow a direct cell-cell contact that might take place in the real physiological condition, it allows positioning the adipocytes and immune cells in close proximity within a dynamic perfusion environment. This configuration, encourages cell-cell signaling as demonstrated by the cytokine expression. Our results are in agreement with those reported by Nitta and Orlando^37^. It was reported that direct adipocyte-immune contact induced higher expression of IL-6 and lower expression of TNF-α as compared to the non-direct contact of adipocyte-immune on the porous membrane.

We envisioned that the microfluidic structure reduces the distances between the two cell types in the co-culture which may enhance more physiologic crosstalk as compared to the transwell-based static culture. These findings are also in agreement with other reports which showed improved cell functions when cells are cultured in a perfused microfluidic chip as compared to static cell cultures^27,36^. These facts favour the on-chip cultures to promote better cell interaction and cell culture environment.

### Study of the on-chip adipocyte-PBMC co-culture’s functions

#### Time-resolved cytokine detection

Using the dynamic on-chip culture, time-resolved measurement of the released cytokines from the same set of cells was examined. The microfluidic chip allowed frequent supernatant sampling without disturbing the cell culture. Data from the time-resolved measurement is expected to be more reliable than the snap-shot measurement in static culture. This dynamic nature of on-chip interaction may emulate the *in vivo* cell interaction with stimuli (or drug) and predict the effect of exposure-/resident-time of stimuli *in vitro*. Additionally, differential cell response can be obtained and compared to baseline values^38^.

**Figure 6:**
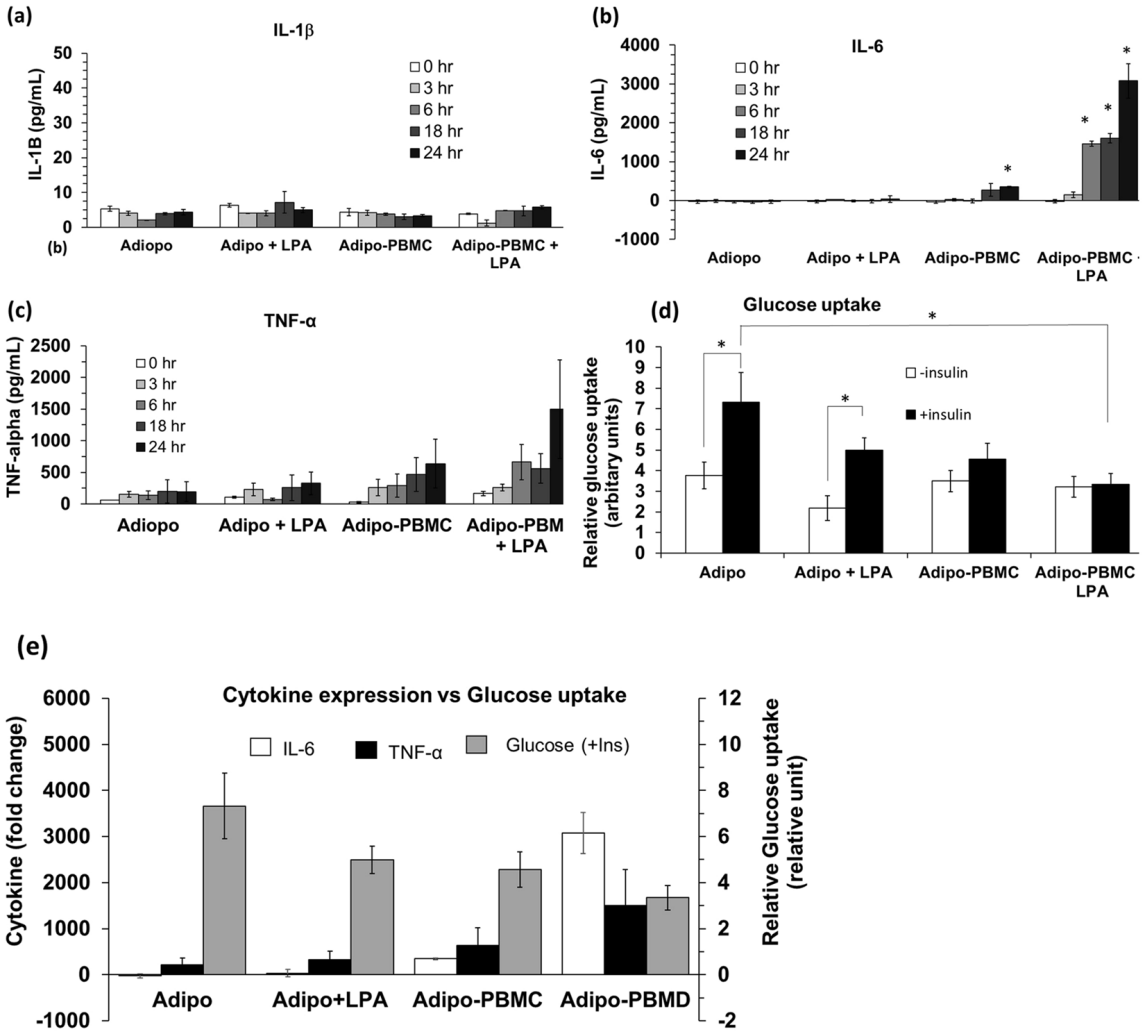
Time-resolved expression of (**a**) IL-1β, (**b**) IL-6 and (**c**)TNF-α and (**d**) glucose uptake measurement at 24 hr obtained from different treatment conditions of cell cultured on-chip. (**e**) Glucose uptake vs Il-6 and TNF-α expression at 24 hr. Data were represented as mean ± SEM. For cytokine experiment, student *t-test* was used for statistical analysis. * Indicates statistical significance at *p* ≤ *0*.*05* as compared to 0 hr. For glucose uptake, student *t-test* and ONE-WAY ANOVA with Bonferroni correction post hoc test was used for statistical analysis. * Indicates statistical difference at *p* ≤ *0*.*05*. All experiments were conducted at least in triplicate.

Adipocyte or Adipocyte/PBMC were cultured on-chip as explained above and the inflammation state was induced by treating the cells with 100 ng/mL of LPA. Supernatants were collected from the immune assay compartment (C) (Fig. 1c) at 0, 3, 6, 18 and 24 hr and IL-1β, TNFα and IL-6 were measured using ELISA. Only a low amount of IL-1β was detected in all culture groups throughout the experiment period (Fig. 6a). Low IL-6 level was also observed throughout the experimental period from untreated and LPA-treated adipocyte mono-cultures (Fig. 6b). In the adipocyte-PBMC co-culture, IL-6 expression was significantly high at 24 hr. IL-6 expression significantly increased in the LPA treated co-culture at 3, 6, 18 and 24 hr with ~3000-fold increase at 24 hr as compared to its baseline value (at 0 hr). Low TNF-α baseline level was seen from untreated, and LPA treated adipocyte mono-cultures (Fig. 6c). Although no statistically significant was observed, TNF-α shows the tendency of gradual increment in untreated adipocyte-PBMC co-culture throughout the 24 hr treatment period. In LPA treated co-culture, TNF-α show tendency of increment at 3, 6 and 18 hr and drastic increased at 24 hr. These data suggest that the time-resolved measurement that offered by the dynamic cell culture provides a clearer prospect of the inflammation development process and paracrine signalling between cells. Long exposure of adipocytes to PBMCs leads to a gradual increase in pro-inflammatory cytokines expression, especially IL-6. LPA treatment enhances acute increment of IL-6 and possibly TNF-α in the co-cultures in which the inflammatory response could be observed as early as 3 hr in IL-6 with the higher response at 6 and 24 hr. (Fig. 6b,c). Altogether, the time-resolved analysis of cytokines using the microfluidic chip provides a useful tool to obtain an in-depth analysis of early diabetes development as well as their potential response to diabetic drugs across different time points.

#### Metabolic status: glucose uptake by adipocytes

We examined the glucose uptake activity of the adipocytes and the effect of insulin treatment on-chip. Cells were co-cultured for 8 hr and treated with 100 ng/mL of LPA for 24 hr and adipocytes were collected for glucose uptake assay using the glucose analogue 2-NBDG glucose assay kit. When treated with 8 μg/mL insulin, adipocytes in mono-culture showed a significant increase in glucose uptake both with and without LPA treatment (Fig. 6d). However, insulin treatment did not induce a significant change in glucose uptake in adipocytes in the co-culture groups as compared to their baselines which may suggest that cells developed insulin resistance condition.

A good correlation between the immune status (the cytokine profile) and metabolic status (glucose uptake) was observed. No cytokines upregulation was detected in adipocyte mono-culture without LPA treatment (Fig. 6a–c), which suggests that the cells did not develop the inflammatory condition. In correlation to the inflammatory profile, a notable increase in glucose uptake was observed from the untreated adipocyte mono-culture upon insulin application (Fig. 6d). It can be interpreted from these data that adipocyte mono-culture displays a normal/healthy adipocyte state where glucose uptake can be stimulated by insulin treatment. Under LPA treatment of adipocyte mono-culture, insulin induced a significant increase in glucose uptake as compared to its baseline. However, there is a tendency of reduction in the insulin-induced glucose uptake as compared to the adipocyte mono-culture without LPA treatment (Fig. 6d). No significant increase in cytokine was observed in the LPA treated adipocyte mono-culture. However, there is an obvious increasing pattern of cytokine, particularly the TNF-α (Fig. 6c) which shows a tendency of increment over time. Thus it can be suggested that 24 hr treatment of LPA to adipocyte did not significantly alter the inflammatory profile and insulin sensitivity of the cell. However, it is anticipated that long-term exposure of adipocyte to LPA may induce inflammation condition and reduce insulin-induced glucose uptake.

Adipocyte-PBMC co-culture without LPA treatment shows a sign of mild inflammation development (Fig. 6b,c). In correlation to the immune status, the metabolic status revealed insulin resistance condition in which insulin failed to induce glucose uptake into adipocytes. Strong inflammation is observed in the LPA-treated adipocyte-PBMC co-culture (Fig. 6b,c). Corresponding to the immune status, insulin did not mediate glucose uptake to adipocytes in this condition (Fig. 6d). In addition, significantly less insulin-induced glucose uptake was observed in the LPA-treated co-culture as compared to the healthy adipocyte mono-culture. These data are consistence with glucose uptake data from human adipocytes which showed reduced insulin-induced glucose uptake in obese and diabetic tissue^36^. Figure 6e summarizes the data obtained in Fig. 6b–d and shows the correlation between the IL-6 and TNF-α expression and the glucose uptake in the adipocytes. The inflammatory and metabolic profiles obtained from these experiments show that the microfluidic adipocyte-PBMC co-culture recapitulates key characteristics of diabetic adipose tissue namely, an inflammatory condition and insulin-resistance characterized by reduced glucose uptake upon insulin treatment.

#### Effect of Metformin and Omega-3 fatty acid

We investigated further the effect of the well-known diabetic drug ‘metformin’ and omega-3 fatty acid on the *in vitro* model. While both non-treated and LPA-treated adipocyte-PBMC co-cultures show insulin resistance characteristic, the LPA-treated group exhibits extremely high inflammatory profile, which may not reflect the physiological condition of type 2 diabetes. The adipocyte-PBMC co-culture without LPA treatment displays mild inflammatory condition which better reflects diabetic physiological condition^39,40^, therefore, it was chosen for these experiments.

**Figure 7:**
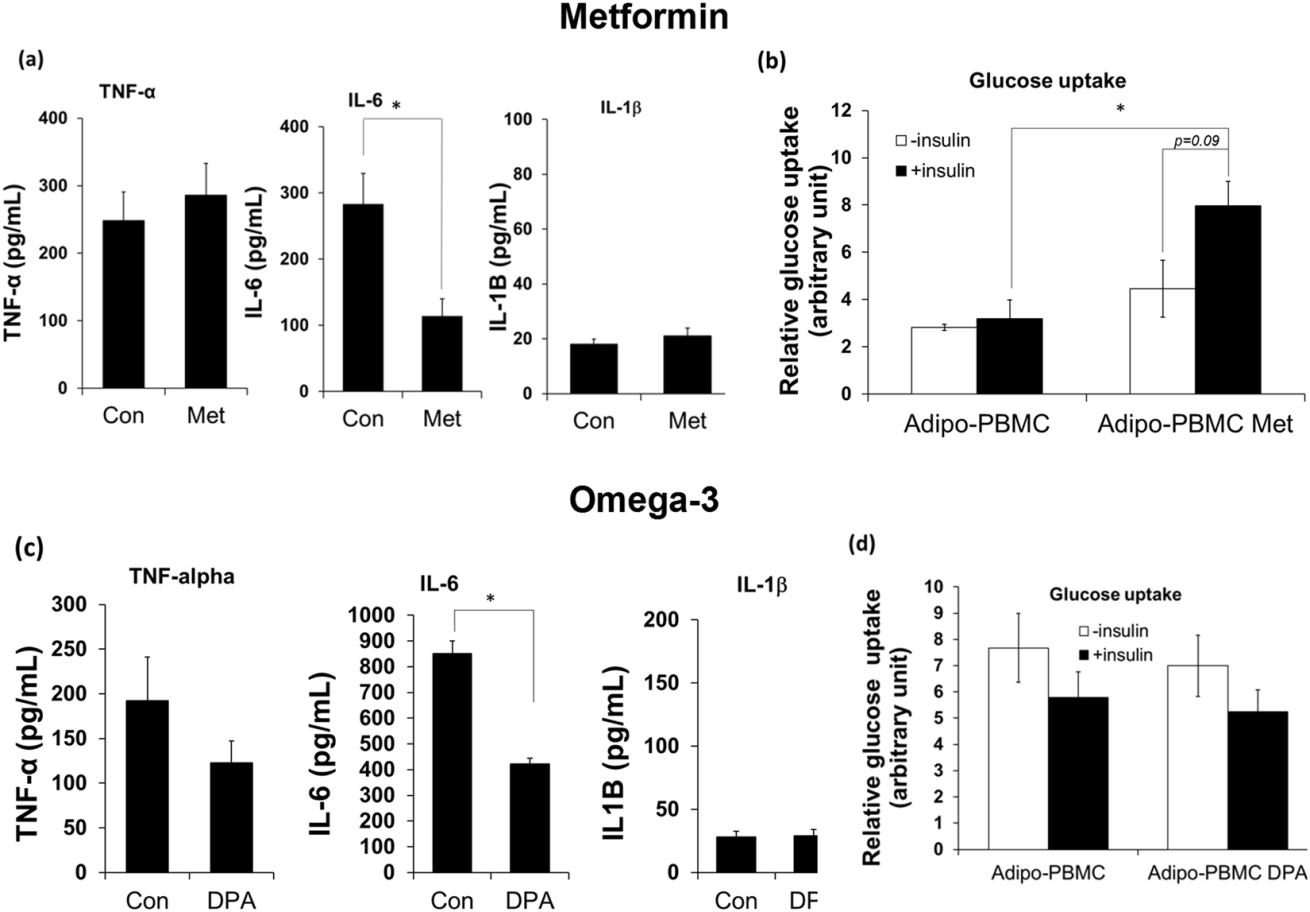
Cytokine expression and glucose uptake obtained from on-chip co-cultures under metformin or neutraceutical treatments. The adipocyte-PBMC co-culture were treated with 10 mM metformin or 20 µM DPA (omega-3) for 24 hr and the supernatant or adipocytes were collected for cytokines and glucose uptake measurement, respectively. (**a**) Expression of IL-1β, IL-6 and TNF-α and (**b**) glucose uptake obtained from control or metformin treated co-cultured cells. (**c**) Expression of IL-1β, IL-6 and TNF-α and (**d**) glucose uptake obtained from control or DPA-treated co-cultured cells. Student *t-test* was used for statistical analysis. * Indicates statistical significance at *p* ≤ *0*.*05*. All experiments were conducted at least in triplicate.

Metformin is known to reduce hepatic glucose production and improve insulin sensitivity^41,42^. To search for the optimal dose of metformin treatment, different concentration of metformin ranging from 2.5–10 mM were tested on adipocyte-PBMC which were cultured in transwell plate. After 24 hr, adipocytes were collected for glucose uptake measurement. While other metformin doses did not show improvement in insulin-induced glucose uptake, metformin at 10 mM tends to improve glucose uptake in the co-cultured cells as compared to its baseline without insulin treatment (*p* = *0*.*08*, Supp. Fig. S6). Therefore 10 mM metformin was chosen for the on-chip testing. Treating the cells with metformin did not induce changes in TNF-α and IL-1β expression level, however it significantly reduced the IL-6 secretion (Fig. 7a). The downregulation of IL-6 may suggest that metformin partially alleviates the inflammatory status of the co-cultured cells which agree with previous reports that revealed metformin as an anti-inflammatory agent in various systems^43,44^. As shown in Fig. 7b metformin was found to increase the insulin-induced glucose uptake (*p* = *0*.*09*) as compared to its baseline non-insulin treatment. In addition, metformin significantly increased glucose uptake in the co-culture cells under insulin treatment comparing to those un-treated with metformin (Fig. 7b). The clear response of the *in vitro* model to metformin treatment confirms the effect of metformin in improving insulin sensitivity in adipocytes as it has been previously reported^45,46^. These results suggest that the *in vitro* model produces supportive results to the previous studies on the static culture system in which metformin alters the inflammatory profile of the cells, i.e. IL-6 and improves insulin-induced glucose uptake in the adipocyte-PBMC culture on-chip. Intriguingly, we observe a tendency of increase in the glucose uptake baseline (without insulin treatment) in metformin-treated cells in both the dynamic model (Fig. 7b) as well as in the static-based culture system (Supp. Fig. S6). Nevertheless, more experimental studies are needed to confirm the role of metformin in improving basal cell activity on glucose uptake.

Similarly, the effect of a neutraceutical compound, omega-3 fatty acid, was tested on the *in vitro* model. Eicosapentaenoic acid (EPA) and docosahexaenoic acid (DHA) are marine origin omega-3 polyunsaturated fatty acids (PUFA). EPA and DHA have been studied extensively and have been shown to improve insulin sensitivity and exerted an anti-inflammatory effect in many diabetic models^47–51^. Docosapentaenoic acid (DPA) is another type of omega-3 PUFA. Due to its low abundant in fish oil and non-availability of the concentrated product, DPA has not been widely studied. It has been shown recently that DPA can effectively improve insulin sensitivity in obese mouse model^52^. Much attention has turned to DPA and its effect on type 2 diabetes. Therefore, DPA was employed for the current study. Adipocyte-PBMC cultured on the chip were treated with 20 µM DPA for 24 hr under perfusion. Afterwards, culture medium and adipocytes were collected for cytokines and glucose uptake measurements, respectively. It is observed from this experiment that the baselines of IL-6 and glucose uptake (Fig. 7c,d) vary notably when compared with the metformin experiment (Fig. 7a,b). We speculated that the difference in the baseline values were due to different PBMC donors. Although all PBMC were taken from ‘healthy control’ subjects, we often observed that different batches of PBMC produced different baselines for cytokines and glucose uptake. Nonetheless, for a set of experiment (i.e. omega-3 treatment or metformin treatment), the batch of PBMC was used for all treatment conditions. Irrespective to the variable in baseline value artefacts, data show that DPA did not alter IL-1β expression level but significantly reduced secretion of IL-6 and a moderately reduced TNF-α secretion (Fig. 7c).

On the other hand, glucose uptake data show no statistical difference in insulin-induced glucose uptake in the DPA treated group. Data obtained from our study suggest that DPA exerts some inhibitory effect on the inflammatory profile however it did not alter insulin sensitivity of the co-cultured cells which agree with data from previous studies^53^ which reported that DPA provides an anti-inflammatory effect to many physiological systems including human. Our data are also in agreement with the previous findings from Oh *et al*.^50^, which reported that omega-3 fatty acid, EPA and DHA, inhibited inflammation induced by the inflammatory agent, lipopolysaccharide (LPS). However, our data oppose other studies^50,52^, which show that omega-3 improved insulin sensitivity in adipocytes from insulin-resistant mouse models. We speculated that the difference in the glucose uptake results might be due to the duration of the omega-3 treatment. In the previous reports^50,52^, the mouse was treated with omega-3 PUFA for ~5–6 weeks whereas in the current study cells were only treated for 24 hr. The short study period in the present study is due to the short viable period of PBMC which only survive for a few days. We speculated that omega-3 might require a longer time to exert its effect on improving insulin sensitivity. Nevertheless, the early sign of anti-inflammation was observed from the on-chip cell cultures. Thus we anticipated that more extended treatment of DPA omega-3 on-chip would eventually lead to improvement in insulin sensitivity.

## Conclusions

An *in vitro* micro-physiological model of the inflamed human adipose tissue has been demonstrated. Human pre-adipocytes were cultured and differentiated on-chip and subsequently co-cultured with human PBMCs to mimic the type 2 diabetic adipose tissue. The dynamic cell co-culture enhanced the cell-cell and cell-stimulus interaction and allowed frequent supernatant sampling without cell culture disturbance. Functional studies showed an over-all agreeable pattern of cytokine profile from the dynamic on-chip and static cultures with relatively stronger and minimal artefact responses observed from the on-chip dynamic cultures. The microfluidic chip allows time-resolved measurement of cytokines that provide reliable data which may give an insight into the tissue inflammation process and consequently the insulin-resistivity development. Good correlation between insulin-resistivity (glucose uptake) and the inflammatory profile was found. Effect of the diabetic drug, metformin, and neutraceutical, omega-3, on the cells cultured on-chip show similar response to the previously reported data including anti-inflammatory effect from both metformin and omega-3 and improve insulin sensitivity from metformin. These data demonstrate that the microfluidic-based *in vitro* model provides a close physiological scenario that exhibits key characteristics of type 2 diabetic adipose tissues and can be used to study the adipogenesis, disease development and diabetic drug screening.

## Materials and Methods

### Materials

Chemicals and reagents were purchased from the following sources: polydimethylsiloxane (PDMS) -Sylgard® 184 from Dow Corning (MI, USA); polyethylene terephthalate (PET) membrane from Corning Transwell (USA); PBS from Biowest (USA); human pre-adipocytes (HPAd) cat # 802s-05a, pre-adipocyte growth medium, adipocyte differentiation medium, adipocyte maintenance medium, and adipocyte starvation medium from Cell Applications (USA); iMDM medium from Thermo Fisher Scientific (USA); Lipid A (LPA) from E.coli serotype R515 (TLR grade) from Alexis Biochemials (SA, USA); TNF-α, IL-6 and IL-8 ELISA kits, CD11b-FITC (clone ICRF44), CD45-PE (clone: 2D1), CD3-PerCP-Cy5.5 (cline: HIT3A), CD19-PE-Cy7 (clone: SJ25C1), CD1c-APC-Cy7 (clone: L161), and CD14-PacBlue (clone: HCD14) from Biolegend (USA); CD56-APC (clone: TULY56) and calceine-AMfrom ThermoFisher Scientific (USA) ;2-NBDG glucose uptake cell-based assay kit from Cayman Chemical (USA); Hoechst 33342 from Molecular Probe (OR, USA); Bradford reagent and 0.4% trypan blue, metformin hydrochloride and docosapentaenoic acid (DPA) from Sigma Aldrich (USA); Other chemicals were of analytical grades from Sigma Aldrich (USA).

### Chip fabrication

The 3D micro-fluidic chip comprises three compartments which fluidically interact through a porous membrane (Fig. 1). The chip was fabricated by assembling the following layers: (i) a glass slide (with dimensions of 25 mm × 75 mm × 1 mm) as a base (ii) Two polydimethylsiloxane (PDMS) layers with thickness of 1 and 3 mm as the fluidic compartments body and (iii) a polyester (PET) membrane with a pore size of 0.4 µm as a barrier between the upper and lower compartments (Fig. 1b,c). The lower part features two circular fluidic compartments, each 6 mm in diameter and 1 mm in depth. The centres of the two compartments are 9 mm apart. The upper compartment which interlaced with the lower compartments has an oval shape with dimensions of 15 mm, 6 mm and1 mm. A 3 mm thick PDMS layer was used to seal the chip and facilitate fluidic tubing connection to the chip.

The fluidic compartments were moulded with PDMS using a poly (methyl methacrylate) (PMMA) mould. The fluidic structure was designed using CorelDraw x6 software (Corel Corporation, Canada) and the PMMA sheets were laser cut using a laser cutter (Universal Laser System, Arizona, USA) to form the mould. The PDMS pre-cursor was mixed thoroughly with the curing agent at a 10:1 ratio and degassed in a negative pressured chamber for ~30 min. The PDMS was carefully poured into the PMMA mould and cured at 60 °C for at least 12 hr. The glass slide and the casted PDMS parts were submerged in isopropyl alcohol (IPA) for 10 min, rinsed with a copious amount of dH_2_O, blown dry with N_2_ gas and cleaned with O_2_ plasma at power 80 for 10 min. After the O_2_ plasma, the lower PDMS part was immediately bonded onto the glass slide and cured at 60 °C for at least 12 hr. The upper and lower PDMS parts and the PET membranes were bonded together using the PDMS precursor. The completed assembled chip was cured at 60 °C for at least 12 hr. The chip was then washed thoroughly with 70% ethanol and exposed to UV light for 30 min. Finally, a copious amount of PBS was flushed through the chip to remove ethanol residual. Each chip contains two independent sets of 3D microfluidic cell culture system for parallel experiments (Fig. 1a).

### Peripheral blood mono nuclear cells preparation

Peripheral blood mononuclear cells (PBMCs) were isolated from blood apheresis cones obtained from the Blood Bank of Health Sciences Authority (Singapore), using Ficoll-Hypaque Plus(Amersham Biosciences, Netherlands) gradient centrifugation. PBMC viability was assessed using trypan blue. Cells were mixed with 0.4% trypan blue at a 1:1 ratio and counted under a microscope. Cell viability was calculated as ([celllive]/[celllive + celldead]) × 100 (%).

### On-chip adipocyte-PBMC dynamic co-culture and treatments

Human pre-adipocytes (HPAd) (Cell Applications, USA) were expanded for 2–4 passages and stored at 0.5 × 10^6^ cells/mL in freezing medium (pre-adipocyte growth medium containing 10% DMSO and 20% FBS) in liquid nitrogen until use. Thawed pre-adipocytes with >85% viability were used in all experiments. 15,000 HPAd cells were inoculated into the lower compartment (B) (Fig. 1c) of the chip and maintained at 37 °C, 5% CO_2_ in humidified atmosphere and supplied with culture medium (pre-adipocyte growth medium) under perfusion with a flow rate of 10 nL/sec for ~3–4 days until reaching confluence. Once the cells reached confluence, the medium was replaced by differentiation medium (Cell Applications cat# 811D-250, USA) to induce adipocyte differentiation. Fully differentiated adipocytes were reached after ~14 days and kept in maintenance medium (Cell Applications cat# 811M-250) until PBMCs inoculation. For adipocyte-PBMC co-culture, the chip with fully differentiated adipocytes was filled with IMDM medium. 10^6^ PMBCs were inoculated into the upper compartment (A) (Fig. 1c). The adipocyte-PBMC cells were maintained in a proper culture condition for ~12 hr without treatment. To induce an inflammatory state, selected sets of adipocytes-immune cells were treated with 100 ng/mL LPA for 24 hr. To demonstrate the *in vitro* model response to drug and nutraceuticals, the cell co-culture was treated with 10 mM metformin or 20 µM docosapentaenoic (DPA), the omega-3 poly-unsaturated fatty acids (PUFA) for 24 hr under perfusion. Control experiment under static condition was also conducted in parallel in transwell insert plates using a similar ratio of adipocyte: PBMC and drug concentrations.

### Cell staining

The cells were fluorescently stained to enhance visualization of the cell morphology. PBMCs were flushed out of the chip and adipocytes were washed with 1x PBS for three times. 2 µM calcein-AM diluted in PBS was applied and incubated for 45 min to stain intracellular content. Cells were washed with 1x PBS for three times and 0.1 µg/mL Hoechst 33342 diluted in PBS was used to stain the nucleus for 5 min. The samples were washed once with 1x PBS to minimize background noise. Upon the completion of staining, the sample was mounted on an upright fluorescent microscope (BX63, Olympus) for capturing of fluorescence images.

### Total lipid content analysis

The lipid content within the differentiated adipocytes was analyzed by utilizing a number of microscopic bright field images. The cell image was captured using an upright optical microscope (BX63, Olympus) which is equipped with a motorized XY stage, a 17.28 megapixel 14-bit CCD camera (DP73, Olympus) and CellSens software (Olympus, Japan). All images were taken by using a 10x objective lens. Once the 2D image was captured, the number of lipid droplet and their size (total area) were estimated by analyzing the image based on the contrast difference between the lipid droplets, which appear darker than the surrounding, and the surrounding background (cell cytoplasm and cell-free surface) and by providing the object filter criteria which can be selectively adjusted in the software.

### Fluorescence-activated cell sorting(FACS) of PBMC & immunofluorescence membrane staining of monocytes

Cells were cultured on-chip (dynamic culture), or trans-well (static culture) for 24 hr and PBMCs were flushed out from the upper compartment of the chip or directly scraped from trans-well insert using cells spatula and analyzed immediately. For FACS analysis, PBMCs were stained with the following anti-human antibodies: CD45-PE for leucocyte, CD3-PerCP-Cy5.5 for T cells, CD19-PE-Cy7 for B cells, CD56-APC for NK cells, CD1c-APC-Cy7 for dendritic cells, CD14-PacBlue for monocytes, and CD11b-FITC for monocytes. All antibodies were diluted at 1:50.

PBMC (1 × 10^6^ cells/100 μl) were incubated in the dark with the specific antibodies for 20 min on ice followed by two washing steps with PBS supplemented with 0.5% FBS and 2 mM EDTA. Afterwards, cells were re-suspended in FACS buffer (PBS supplemented with 0.5% BSA, 2 mM EDTA and 0.1% sodium azide) and analyzed on FACSCanto (BD, Germany) with BD FACSDiva Flow Cytometry Software (BD Biosciences). Compensation was done with UltraComp eBeads Compensation beads (Invitrogen, ThermoFischer, USA). For gating, FMO (Fluorescence minus one) served as control. Data were analyzed using FlowJoTM v10 software (TreeStar, Inc. Ashland, USA).

For staining of monocyte on the membrane, the chip was disassembled, and the membrane was carefully detached from the chip and placed in a 24 well plate. The membrane was blocked with 2% BSA in PBS for 30 min, and monocytes were stained using 1:100 FITC-CD11b for 2 hr at room temperature. The membrane was washed with PBS, and the cell nucleus was stained using 1:500 Hoechst for 5 min. The membrane was then washed thoroughly with PBS, and the fluorescence signal was observed under a microscope (BX63 upright microscope, Olympus, USA). Hoechst and CD11b positive cells were identified as monocytes.

### Cytokine immunoassay

Enzyme-linked immunosorbent assay (ELISA) was used to detect the inflammatory cytokines released from the co-cultured cells. The supernatant was collected from the immune assay compartment (C) (Fig. 1c) at different time intervals, i.e. 0, 1, 3, 12 and 24 hr, after the desired treatments. To collect the supernatant, the perfusion was stopped, and 20 µL of fresh medium was gently injected into the chip inlet. The injected fresh medium forced equivalent amount of supernatant (20 µL) to overflow out of the immunoassay compartment (C) outlet. The overflown medium was collected and stored at −20 °C until use. The chip was connected back to the perfusion circuit to continue the cell culture. For control experiments in static culture, the supernatant was aspirated directly from the transwell plate and stored at −20 °C until use. Three inflammatory cytokines, namely, TNF-α, IL-6 and IL-1β, were targeted in this study. Samples collected from the chip were diluted at 1:5 ratio with fresh iMDM medium. 35 µL of the diluted sample was used for each ELISA measurement. As larger volume of culture media was used for static culture, supernatant collecting from the static cultures were diluted at different dilution to ensure that ELISA signals can be captured while signals would not reach saturation. Supernatant samples collected from static culture conditions were diluted at 1:1 and 1:3 for IL6 and IL-1β, respectively, whereas non-diluted samples were used for TNF-α ELISA measurement. ELISA experiments were conducted according to the manufacturer protocol. 5 µg/mL of capture antibody was immobilized onto a 96 well plate and kept at 4 °C overnight. Tris buffer saline (50 mM Tris and 150 mM NaCl, pH 7.4) with 0.1% tween 20 (TBST) was used as washing buffer between each ELISA step. The plate was blocked with 2% BSA in PBS for 30 min, and samples, as well as standards, were incubated with the immobilized capture antibody for 2 hr. 5 µg/mL of detection antibody and 1:1000 horse radish peroxidase (HRP) solution were then sequentially applied to the ELISA plate and incubated for 1 hr. and 30 min., respectively. Finally, TMB solution was applied and incubated for ~5–10 min until the reaction sample turns blue. The reaction was stopped using 2 M H_2_SO_4_, and the optical signals were measured at 450 nM using Enspire® Multimode Plate Reader (PerkinElmer, USA).

### Glucose uptake assay

Glucose uptake assay was performed using fluorescent-labelled deoxyglucose 2-deoxy-2-[(7-nitro-2,1,3-benzoxadiazol-4-yl) amino]-D-glucose or 2-NBDG as glucose analogue probe. Adipocytes were starved for 1 hr in adipocyte starvation medium and 15 min in PBS. 150 µg/mL 2-NBDG diluted in KRPH buffer [20 mM HEPES, 5 mM KH2PO4, 1 mM MgSO4, 1 mM CaCl2, 136 mM NaCl, 4.7 mM KCl, pH 7.4] with 2% BSA and 8 µg/mL insulin was then applied to adipocytes and incubated for 1 hr. Adipocytes were washed with copious amount of PBS and lysed using lysis buffer [50 mM Tris, 150 mM NaCl, 1% Triton-X100, pH 8] for 20 min at room temperature. Lysates were diluted at 1:4 and 1:9 with PBS for fluorescence and total protein measurements. To measure total protein content, 5 μL of sample was incubated with 250 μL of Bradford reagent for 5–10 min at room temperature until the reaction changed from brown to blue.

2-NBDG fluorescence signal was detected at excitation/emission wavelength of 485/535 nm and Bradford chromogenic assay was detected at 595 nm using Enspire® Multimode Plate Reader (PerkinElmer, USA). Data was represented as a relative unit where the fluorescence intensity signal (a.u.) of glucose uptake was normalized with total protein content (µg/mL).

## Supplementary information

Supplimentay materials
