# Supplementary material for: In vitro micro-physiological model of the inflamed human adipose tissue for immune-metabolic analysis in type II diabetes
Source: Sci Rep. 2019 Mar 20;9:4887. doi: 10.1038/s41598-019-41338-3 (PMC6426956; doi:10.1038/s41598-019-41338-3)
Supplement: Supplementary file 1 — Supplimentay materials [file 41598_2019_41338_MOESM1_ESM.pdf]

Supplementary Information

In vitro micro-physiological model of the inflamed human adipose tissue for immune-metabolic analysis in type II diabetes

Patthara Kongsuphol[a], Shilpi Gupta[b], Yunxiao Liu[a], Sajay Bhuvanendran-Nair-Gourikutty[a], Subhra K. Biswas[b] and Qasem Ramadan[a]*

(a) (b)

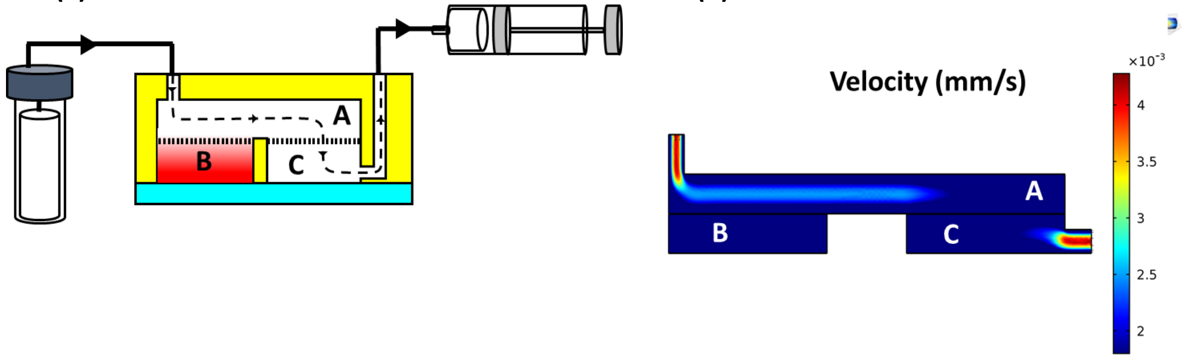

(c)

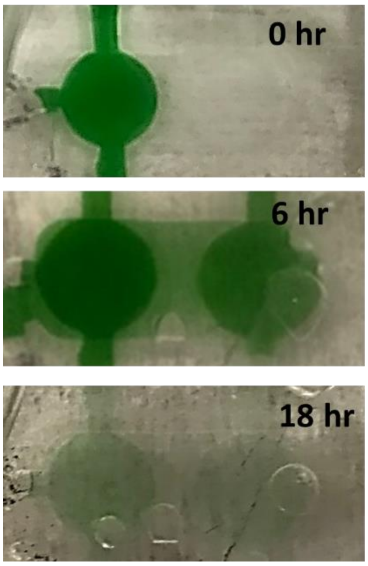

Figure S1: (a) Cross-sectional drawing of the microfluidic chip with the three compartments. (b) Finite Element Analysis of the fluid velocity profile through the microfluidic chip at a flow rate of 10 nL/s. (c) The lower compartment (B) was filled with green dyed PBS solution while the other compartments were filled with clear (undyed) PBS. After 18 hr of perfusion at 10 nL/sec, the coloured solution completely vanished from the compartment B. This indicates that the dye diffused into the upper compartment (A) and washed out by perfusion through the outlet in compartment (C). Meanwhile, the clear PBS diffused to the lower compartment (B) and slowly replaced all the dyed solution in the compartment.

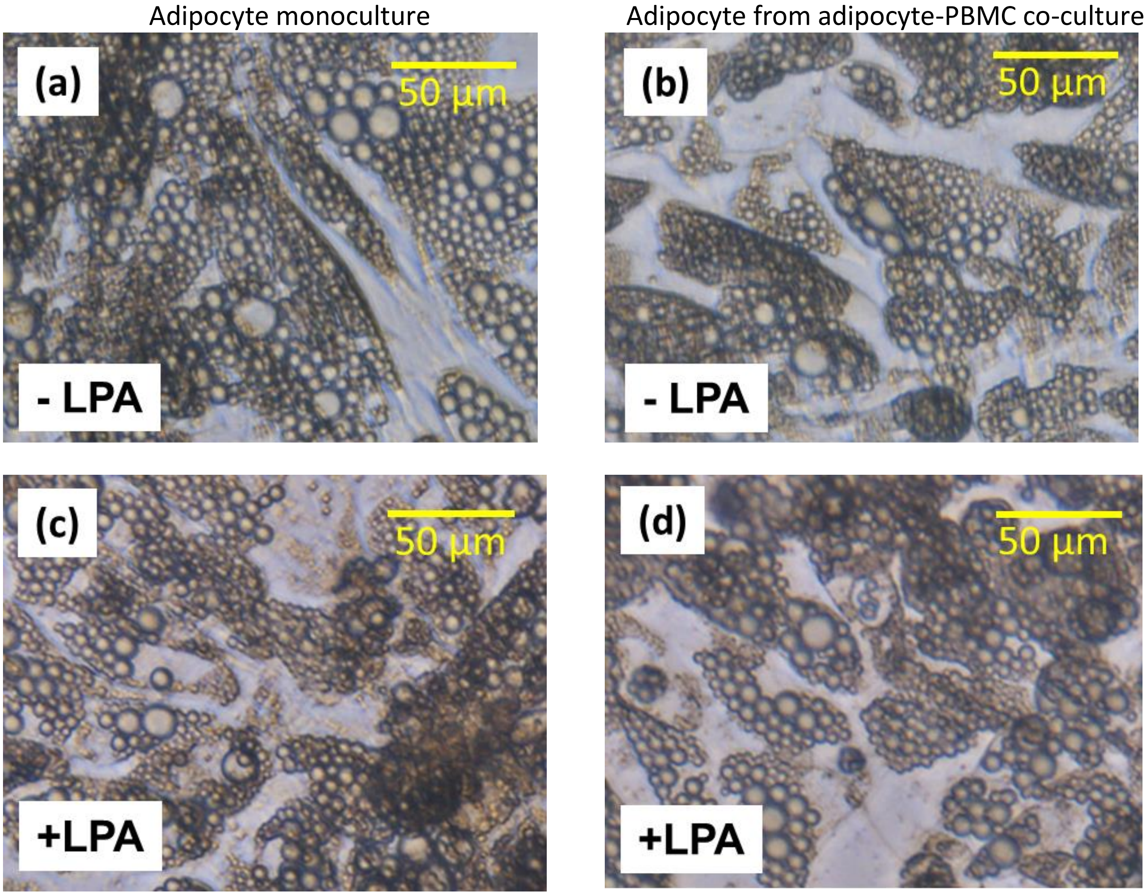

Figure S2: No significant changes in droplet size were observed in the static cultures where cells exhibited similar morphology in all treatment groups

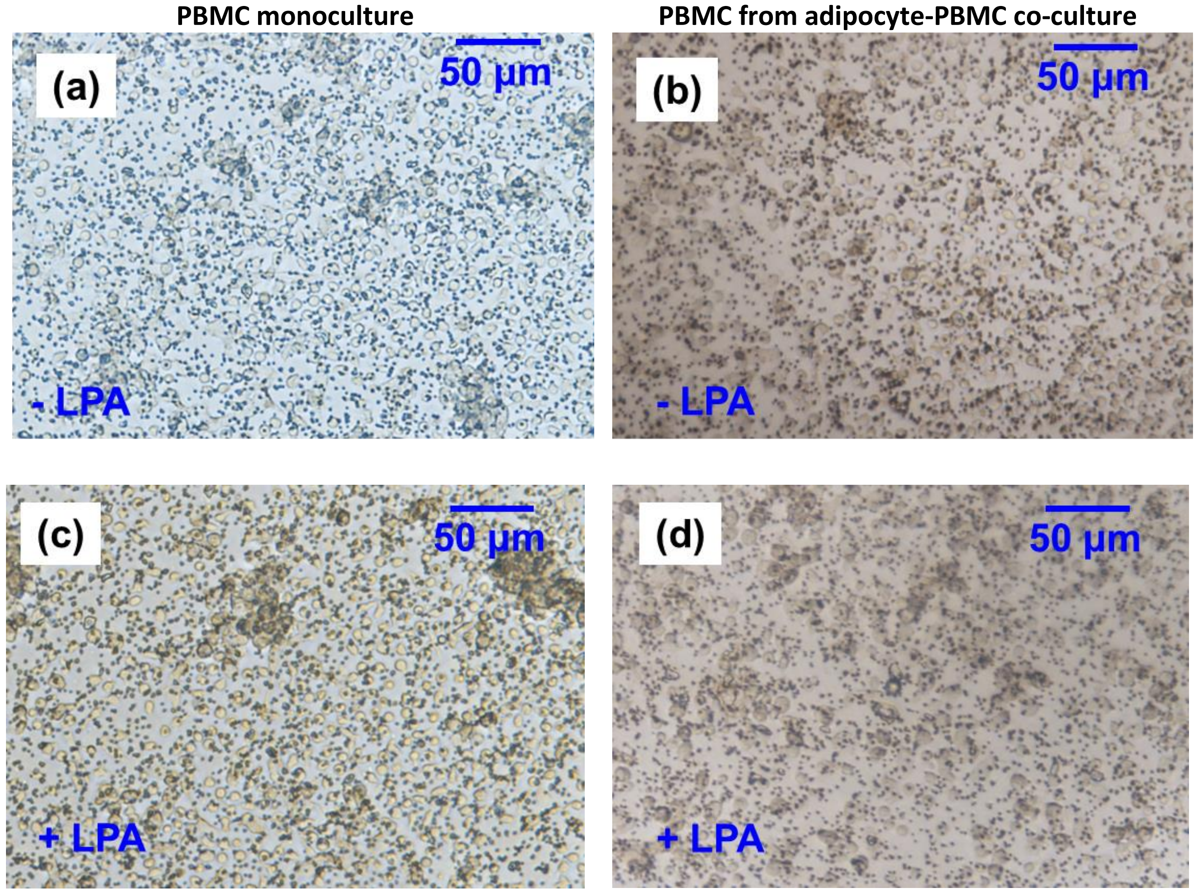

Figure S3: Adipocyte-PBMC co-cultured on chip

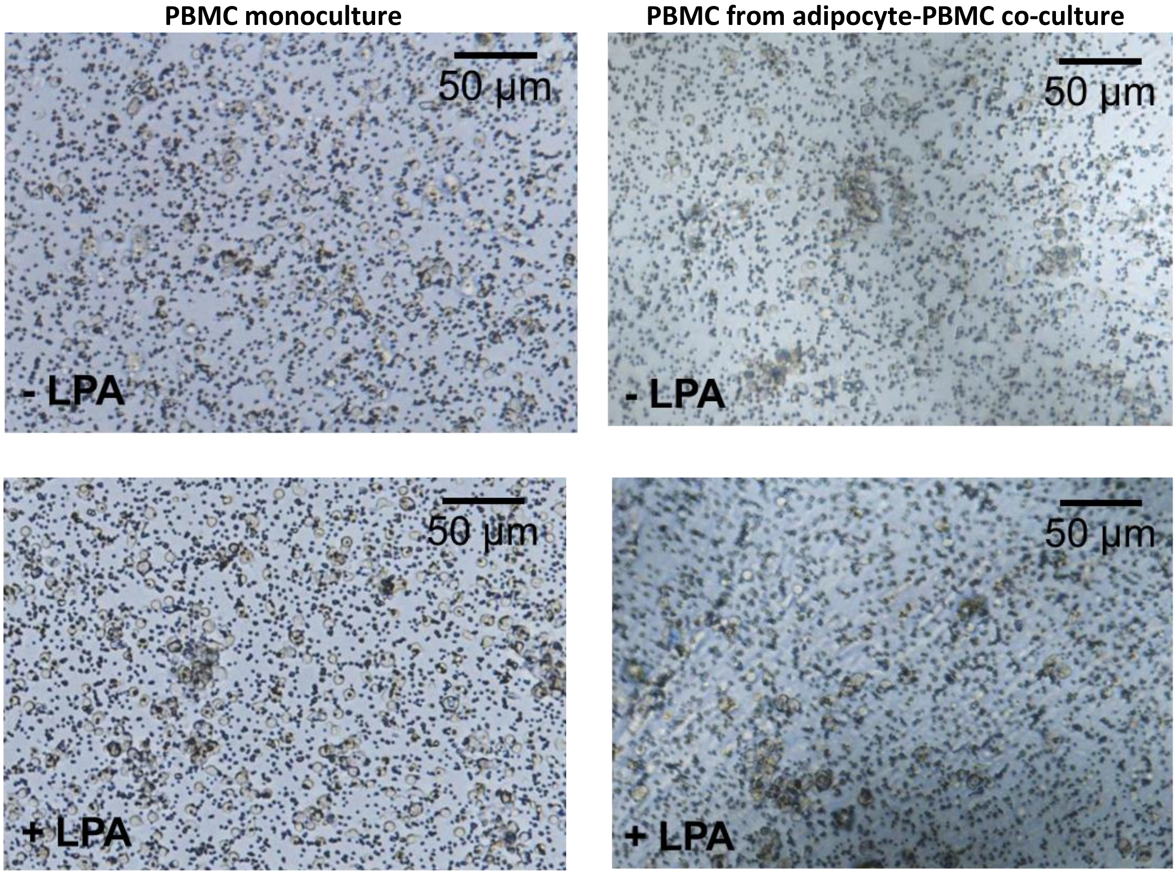

Figure S4: Adipocyte-PBMC static co-culture on trans-well plate

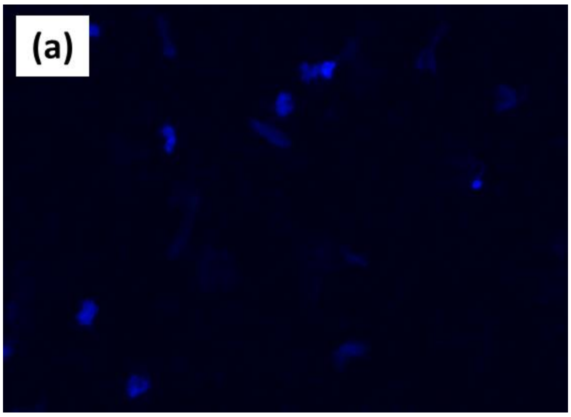

Hoechst (nucleus)

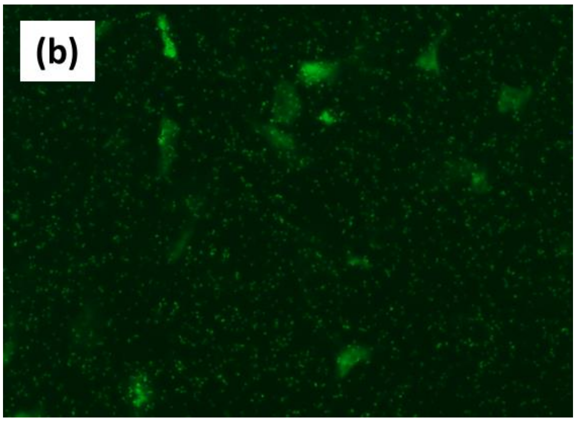

CD-11B-FITC (Monocytes)

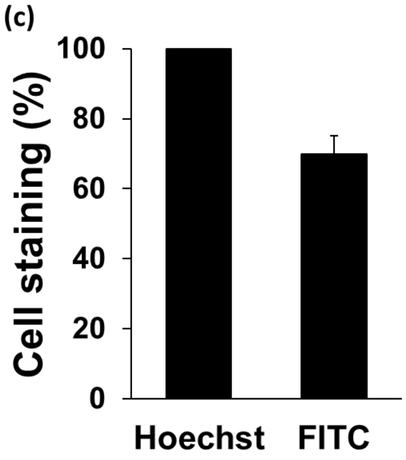

Figure S5: (a) Fluorescence image of stained PBMC nucleus (Hoechst) that adhered onto the chip’s membrane after washing. (b) Fluorescence image of monocytes (CD-11B-FITC positive) that adhered on the membrane. (c) Percentage of monocytes (CD-11B-FITC positive cells) as compared to all nucleated cells (Hoechst) remained on the membrane.

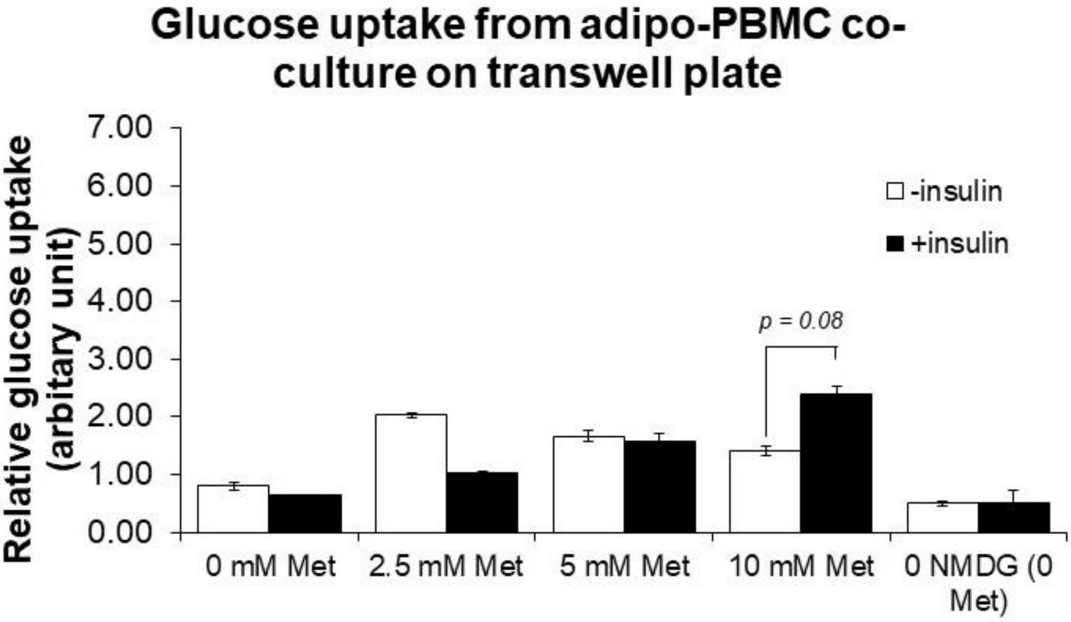

Figure S6: Glucose uptake in metformin-treated cells in the static-based culture system.
